# ROLE OF ARTERIOVENOUS VASCULAR LOOPS IN MICROSURGICAL RECONSTRUCTION OF THE EXTREMITIES

**DOI:** 10.1590/1413-785220182602187220

**Published:** 2018

**Authors:** GUSTAVO BERSANI SILVA, BRUNO AZEVEDO VERONESI, LUCIANO RUIZ TORRES, RAQUEL BERNARDELLI IMAGUCHI, ALVARO BAIK CHO, HUGO ALBERTO NAKAMOTO

**Affiliations:** 1. Hand and Microsurgery Group, Instituto de Ortopedia e Traumatologia, Hospital das Clinicas HCFMUSP, Faculdade de Medicina, Universidade de São Paulo, São Paulo, SP, Brazil.

**Keywords:** Arteriovenous fistula, Microsurgery, Upper extremity, Lower extremity., Fístula arteriovenosa, Microcirurgia, Extremidade superior, Extremidade inferior.

## Abstract

**Objective:**

To analyze 10 consecutive cases of microsurgical arteriovenous loops created to reconstruct complex injuries from March 2011 to May 2012.

**Methods:**

This observational cohort-type study conducted by the Hand and Microsurgery Group at the HC-FMUSP included patients who were candidates for microsurgical reconstruction as a last alternative to amputation of the limb with proven absence of adequate recipient vessels for primary microsurgical anastomosis, in a prospective and consecutive manner. We analyzed 14 variables (epidemiological, clinical, procedure-related, and outcome) in patients who underwent reconstruction using an arteriovenous loop utilizing a single-stage or two-stage procedure.

**Results:**

The injuries were mostly traumatic (80%). The success rate of the single-stage procedure was 75%, and 17% for the two-stage procedure. The rate of preservation for the injured limb was 44%.

**Conclusion:**

This study reinforces the more recent understanding that the indication for single-stage or two-stage reconstruction should be individualized; our findings favor the single-stage reconstruction. This technique should be used in selected cases, as a last reconstructive alternative before amputation, and further studies are necessary to confirm its safety and efficacy in our practice. Level of Evidence IV; Case series.

## RESUMO

**Objetivo:**

Analisar dez casos consecutivos de alças vasculares microcirúrgicas realizadas para reconstrução de feridas complexas no período de março de 2011 a maio de 2012.

**Métodos:**

Estudo de observação*,* analítico do tipo coorte, realizado pelo Grupo de Mão e Microcirurgia do Instituto de Ortopedia e Traumatologia do HC-FMUSP. Foram incluídos, prospectivamente e de maneira consecutiva, os pacientes candidatos à reconstrução microcirúrgica como última alternativa à amputação do membro, com comprovada ausência de vasos receptores adequados para anastomose microcirúrgica primária. Foram analisadas 14 variáveis (epidemiológicas, clínicas, relacionadas ao procedimento e resultados) nos pacientes submetidos à reconstrução com alça em tempo único e em dois tempos.

**Resultados:**

As lesões foram de etiologia traumática em sua maioria (80%). A taxa de sucesso do procedimento em tempo único foi de 75%, e em dois tempos de 17%. A taxa de manutenção do membro lesado foi de 44%.

**Conclusão:**

Este estudo reforça o entendimento mais recente de que a indicação de reconstrução em um ou dois tempos deve ser individualizada, e nossos resultados favorecem a reconstrução em tempo único. A técnica deve ser usada em casos selecionados, como última opção reconstrutiva à amputação, sendo necessários mais estudos para atestar sua segurança e eficácia em nosso meio. Nível de Evidência IV; Série de casos.

## INTRODUCTION

Tissue transfer through reconstructive microsurgery is an important therapeutic option for treating complex injuries resulting from trauma, infection, or cancerous infiltration of the limbs. However, some patients have receiving vessels of very poor quality near the wound area, thus impeding the transference of a free flap to treat the defect. In these cases, the necessity of the flap’s vessels to communicate with healthy recipient artery and veins demand techniques which permit the use of receiving vessels far from the wound site, including microsurgical arteriovenous loops.^1^ ^,^ ^2^

The concept of the microsurgical vascular loop, introduced by Threlfall et al.^3^ in 1982 and popularized by Grenga starting in 1987,^1^ is a useful and versatile tool for facilitating transfers of tissue to receiving areas which lack adequate vessels for microsurgical anastomosis. The technique involves creating an arteriovenous fistula with a vein graft, usually the great saphenous vein. The midpoint of the fistula, which is located near the area to be reconstructed, is then sectioned to provide vessels for arterial inflow and venous drainage for the microsurgical anastomoses.^1^ ^-^ ^4^ This consequently constructs a system with high flow and low resistance near the injury, offering good quality vessels to obtain free flaps.^5^ ^,^ ^6^

Two options can be used to extend the receiving vessels of a free flap: interposition of a vein graft or the creation of an arteriovenous loop.^7^ Several studies have shown the superiority of the vascular loop over long venous grafts with regard to the risk of thrombosis and need for reinterventions.^8^ ^-^ ^12^

Despite its proven clinical applicability, some details of the surgical technique are still the subject of controversy, such as creating the free flaps during the same surgery (in one single-stage procedure) or later, after the construction and maturation of the arteriovenous shunt (surgery in two stages at two different times), highlighting the need for better understanding of the different variables involved in this technique.^2^ ^,^ ^4^ ^-^ ^7^

Currently there are no clear criteria which support the decision to perform surgery in one or two stages, and the severity of the injury and patient characteristics (namely the ability to tolerate major surgery due to comorbidities or difficulty obtaining clinical stabilization) are factors that traditionally guide decision-making.^2^

These controversies led us to conduct a prospective analysis of 10 consecutive vascular loops used to reconstruct complex wounds in limbs which did not have receiving vessels.

## MATERIALS AND METHODS

From March 2011 to May 2012, 10 microsurgical vascular loops were created in 9 patients evaluated prospectively.

The study was approved by the institutional review board under process number 1083. All patients signed an informed consent form.

The criteria for inclusion in this study were:

Patients presenting with complex wounds (exposure of the bone, tendon, or vascular-nervous bundle) in the limbs, candidates for microsurgical reconstruction as a last alternative to amputation;

Imaging exams (computed angiotomography/magnetic resonance angiography) or intraoperative assessment indicating inadequate vessels for microsurgical anastomosis (inadequate flow in arterial trunks near the injury to be rebuilt);

Minimum follow-up of two months after creation of the loop.

The following variables were assessed: sex, age, comorbidities, etiology of the injury, whether loop involved 1 or 2 stages, number of days between AV loop creation and definitive flap elevation (when loop involved 2 stages), flap used, artery receiving the loop, use of the ipsi- or contralateral saphenous vein, loop success, rate of reoperation, whether the limb was saved or not, which anticoagulant drug was used in the postoperative period, and complications.

The surgical technique employed to create the loops used the contralateral great saphenous vein (8 loops) or the ipsilateral great saphenous vein (2 loops). End-to-side anastomoses to the femoral artery (9 loops) or popliteal artery (1 loop) were employed and end-to-end anastomoses of the contralateral saphenous vein to the ipsilateral vein were used when necessary (contralateral saphenous vein graft). All the loops were anastomosed after filling with a heparin solution (20 IU/mL) and all patients used ASA (200mg/day) and hyperhydration during the postoperative period to prevent clotting and vasospasm, respectively.

For the statistical analysis, we used SPSS version 20.0 software (SPSS Inc, Chicago, IL, USA) and performed descriptive statistical and univariate analysis using Fisher’s exact test, comparing the groups of vascular loops created during 1 or 2 procedures. A P-value <0.05 was considered statistically significant. The groups had similar age distribution.

## RESULTS

**Figure 1:**
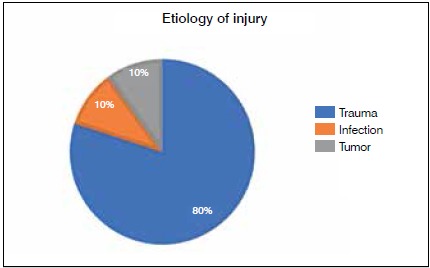
Etiology of injury.

The study included eight men and one women, with ages ranging from 21 to 48 years (mean: 33.7 years). The patients were followed for a mean period of 7.9 months (minimum of 2 and a maximum of 14 months). Most of the injuries were traumatic (7 cases); in the other cases, the etiology was chronic osteomyelitis and tumor (squamous-cell carcinoma) with one case each, respectively. (Figure 1)

**Figure 2:**
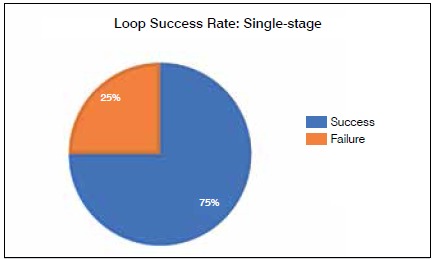
Success rate for loops created in single-stage procedure.

**Figure 3:**
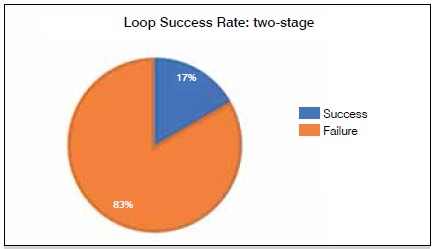
Success rate for loops created in two-stage procedure.

Four reconstructions were performed in a single-stage procedure, and six were carried out in two stages. The average time between the time when the loop was created and coverage with the microsurgical flap was 2.6 days in the cases where the two-stage procedure was used. The success rate for the one-stage procedure was 75%, (Figure 2) and 17% for the two-stage procedure. (Figure 3)

**Figure 4:**
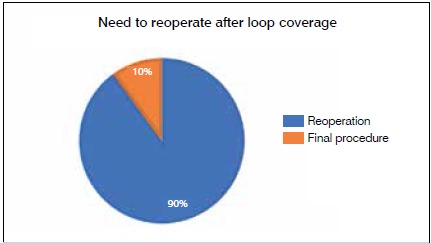
Need to reoperate after surgical skin coverage using vascular loop.

The rate of reoperations after loop reconstruction was 90% (Figure 4), considering re-explorations of the anastomosis, the need for new flaps, or amputation of the limb. The salvage rate for the injured limb was 44% (4/9).

The data are summarized in Table 1.

**Table 1:** Variables analyzed.

Patient	Sex	Age	Etiology	Comorbidities	Months of follow-up	Time of Loop	Reoperation	Success of Loop	Limb saved	Post-op	Complications	Coverage Used	Recipient Artery	Contralateral saphenous vein
1	M	33	TR		14	2	No	Yes	Yes	ASA		Latissimus dorsi	Femoral	Yes
2	M	24	TR		13	0	Yes	Yes	No	ASA		ALT	Femoral	Yes
3	M	38	TR	HBP	12	5	Yes	No	Yes	ASA		Gastrocnemius M and L	Femoral	Yes
4	M	27	TR		10	0	Yes	No	Yes	ASA		ALT + Cross-leg	Femoral	Yes
5	M	44	TR		9	7	Yes	No	No	ASA		Vacuum dressing	Femoral	Yes
6	M	21	TR		8	0	Yes	Yes	No	ASA		Ectopic reimplantation	Femoral	No
7	M	32	TR		4	6	Yes	No	New loop	ASA		Vacuum dressing	posterior tibial	No
8	M	32	TR		4	5	Yes	No	No	ASA		Vacuum dressing	Femoral	Yes
9	M	38	TU		3	1	Yes	No	No	ASA		Vacuum dressing	Femoral	Yes
10	F	48	I		2	0	Yes	Yes	Yes	ASA		Rectus femoris	Femoral	Yes

**Figure 5:**
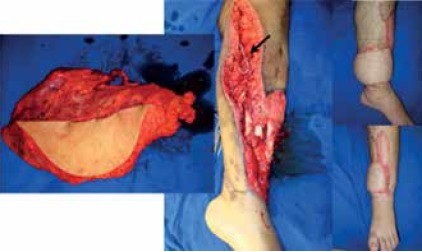
Example of reconstruction using loop created in two-stage procedure.

**Figure 6:**
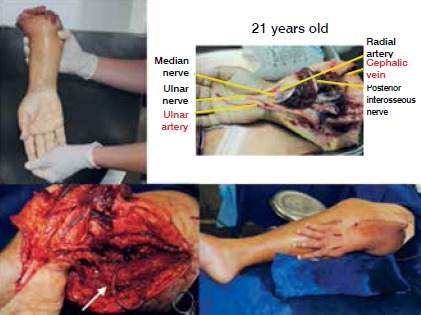
Example of reconstruction using loop from single-stage procedure.

Figure 5 illustrates one case of reconstruction in two stages, while Figure 6 demonstrates the versatility of using the vascular loop during a single-stage procedure.

Severe trauma in lower left leg. Angiotomography showed only the posterior tibial artery was patent. Latissimus dorsi flap used to cover the leg injury, rebuilt with vascular loop created in two-stage procedure. Arrow: Vascular loop constructed 2 days prior to coverage with the flap. Right: 12 months after surgery, patient walking normally.

Traumatic amputation of the right forearm. Vessels in good condition were identified in the amputated portion, but receiving vessels in the arm were not suitable for anastomosis. Construction of an arteriovenous loop with the ipsilateral saphenous vein for ectopic reimplantation of the amputated limb. Arrow: Microsurgical vascular loop. Finalized ectopic reimplantation, with good perfusion.

No statistically significant difference was observed between the groups with vascular loops made during one or two stages in relation to the flap success rate (p=0.19) or limb salvage rate (p=1.0).

## DISCUSSION

In Brazil, as in many developing countries, limb amputation imposes such a severe social stigma and functional limitations due to the lack of suitable prosthesis, that the surgeon is often impelled to manage dramatic cases in which microsurgical reconstruction of a limb lacking good quality recipient vessels is the only suitable treatment alternative. This unique study in the national literature sheds light on this peculiar situation and evaluates the role of the vascular loop as an auxiliary technique in treating these challenging injuries.

Several authors defend creating vascular loops in two stages due to the theoretical advantages obtained after vein arterialization (when there is less chance of collapse and increased vessel diameter) compared to creating the loop and raising the flap in a single stage. They argue that using two stages reduces the chance of complications related to “serial” anastomoses, permitting complications in the loop to be seen before the flap is transferred.^13^ However, recent studies have challenged this concept, and attempt to establish clearer criteria for using a one- or two-stage procedure.^2^ ^,^ ^14^ ^-^ ^16^

In our study, the success rate for free flaps using the vascular loop created in one stage was 75% and limb salvage was 50%, better results than those obtained from using a vascular loop created in two stages, which had a flap success rate of 17% and limb salvage rate of 33%. Despite the better results for the single-stage procedure, no statistically significant difference was observed, which may be justified by the small number of cases.

Cavadas^2^ rationalized indicating the two-stage procedure in special cases, such as:

Patients with severe (non-cardiac) comorbidities who cannot tolerate major procedures Problems with a loop constructed during a single-stage procedure, such as intraoperative identification of significant atheromatosis of the receiving vessels or thrombosis of the shunt, with the loop created and patient monitored to assess loop patency, creating the flap in ideal conditions.^2^

Prospective analysis of data permitted objective evaluation of 14 variables, studying the behaviour of vascular loops made during one- or two-stage procedures. The correct approach should consider the extent of the injury and the characteristics of the patient, such as their ability to support reconstruction in a single stage, nonetheless intraoperative assessment of the proximal limb vessels and loop conditions are also important, since we would rather abort the reconstruction and perform the procedure in two stages than subject the patient to flap coverage under less than ideal conditions.

Besides indication, rigorous implementation of the arteriovenous loop construction techniques also contributes to the success of the procedure. The authors favour AV loops created with an arterial end-to-side anastomosis and end-to-end venous anastomosis when necessary. Special attention should be paid to filling the graft with heparin before starting the anastomoses to prevent twisting of the long vein graft. It is also important to carefully prepare the tissue bed where the loop will rest during the two-stage reconstruction in order to prevent kinking or compression of the loop in the subcutaneous tissue and consequent thrombosis.

## CONCLUSIONS

This study reinforces the more recent understanding that indication for single-stage or two-stage reconstruction should be individualised, and our results favour single-stage reconstruction. The technique should be used in selected cases, as a last reconstructive option to amputation, but more studies are needed to attest to its safety and efficacy.
